# BDNF Gene Polymorphism and Antidepressant Response in Han Chinese Patients with First-Episode Late-Life Depression

**DOI:** 10.31083/AP39955

**Published:** 2025-04-24

**Authors:** Han Wu, Jiao-jiao Zhou, Xue-yan Chen, Dan-di Zhu, Feng Bao, Wei Zheng, Li Ren, Wei-gang Pan, Chao-meng Liu

**Affiliations:** ^1^Beijing Key Laboratory of Mental Disorders, National Clinical Research Center for Mental Disorders & National Center for Mental Disorders, Beijing Anding Hospital, Capital Medical University, 100088 Beijing, China; ^2^Department of Psychiatry, The Affiliated Brain Hospital of Guangzhou Medical University, 510370 Guangzhou, Guangdong, China; ^3^Advanced Innovation Center for Human Brain Protection, Capital Medical University, 100088 Beijing, China; ^4^Peking University Huilongguan Clinical Medical School, Beijing Huilongguan Hospital, 100096 Beijing, China

**Keywords:** antidepressants, depression, mood disorders

## Abstract

**Objective:**

This study investigated the association between brain-derived neurotrophic factor (BDNF) gene polymorphisms and antidepressant response in patients with first-episode late-life depression (LLD).

**Methods:**

A total of 72 patients with first-episode LLD were recruited and 57 completed an 8-week course of antidepressant treatment. Participants were assessed at baseline and post-treatment using the 17-item Hamilton Depression Rating Scale (HAMD-17) and the Repeatable Battery for the Assessment of Neuropsychological Status (RBANS). Serum BDNF levels were measured via Enzyme-Linked Immunosorbent Assay (ELISA) and BDNF gene polymorphisms were genotyped using the Agena® MassARRAY system.

**Results:**

After 8 weeks, 17 of the 57 patients with LLD showed effective treatment response (effective group), while 40 were classified as ineffective. Significant post-treatment improvements were observed across the cohort in HAMD-17 and RBANS scores, and serum BDNF levels compared with baseline (*p* < 0.05). However, the effective and ineffective groups did not have significantly different RBANS scores or serum BDNF levels (*p* > 0.05). Binary logistic regression identified male sex (OR = 10.094, *p* = 0.007) and BDNF gene polymorphism (OR = 6.559, *p* = 0.003) as predictors of treatment efficacy.

**Conclusion:**

Antidepressant treatment for 8 weeks altered serum BDNF levels in patients with LLD, with male patients carrying the *Val/Val *genotype potentially responded better to conventional antidepressants. The small sample size may limit the generalizability of these findings.

**Clinical Trial Registration:**

The study was registered at https://www.chictr.org.cn (registration number: ChiCTR1900024445).

## Main Points

1. After 8 weeks of antidepressant treatment, Han Chinese patients with first-episode LLD exhibited significant improvements in depressive symptoms, increased serum BDNF levels, and enhanced cognitive function.

2. This study found no significant correlation between changes in depressive symptoms and alterations in serum BDNF levels or RBANS scores.

3. Male patients with LLD who possess the *Val/Val *genotype potentially respond better to conventional antidepressant therapy.

## 1. Introduction

Late-life depression (LLD), affecting individuals aged 60 years and older, is prevalent in approximately 10–15% of community-dwelling older people [1, 2]. This prevalence rate increases in those with concomitant physical conditions, leading to diminished quality of life, impaired social functioning, and a heavier burden on caregivers [3, 4]. Apart from typical depressive symptoms, it often manifests with cognitive impairments, severely affecting treatment efficacy, social functioning, and overall quality of life. Besides typical depressive symptoms, cognitive impairments are common in LLD, severely impacting treatment outcomes, social functioning, and overall quality of life. The prognosis for LLD is worse than that for younger adults [5, 6]. Follow-up studies indicate that 44% of patients with LLD experience a fluctuating course of remission and relapse, 32% progress to chronic depression, and only 23% achieve favorable outcomes [7]. While continuation of antidepressant therapy has similar efficacy in older and younger adults [8], over half of the patients with LLD in remission relapse, primarily within 2 years [9]. Consequently, identifying objective markers for treatment response and prognosis in older patients is critical to guiding more effective interventions.

Emerging research suggests that alterations in brain-derived neurotrophic factor (BDNF) are associated with depression onset [10, 11]. BDNF, a critical neurotrophic factor in the brain, supports neuron survival, differentiation, and growth. It plays a protective role, promoting neuronal repair and regeneration after injury [12]. The BDNF gene, located on the short arm (p13 region) of chromosome 11, includes several polymorphic sites, with the *G196A* polymorphism drawing particular attention [13]. This polymorphism occurs within a functional coding region, where a guanine (*G*) nucleotide is replaced by adenine (*A*). This substitution changes the BDNF precursor peptide, where valine (*Val)* is replaced by methionine (*Met*) at position 66, resulting in the BDNF *Val66Met* polymorphism [14, 15]. Although this substitution does not alter the function of mature BDNF protein, it disrupts the intracellular processing and secretion of BDNF precursors. The presence of the Met allele interferes with normal maturation and secretion of BDNF, potentially influencing the onset, progression, and outcome of depressive disorders [16, 17].

The BDNF *Val66Met* polymorphism is associated with brain structure and mood disorders [18, 19]. Research suggests that the Met allele increases vulnerability to dysfunctions in the uncinate fasciculus, a neural tract involved in negative emotional processing, memory deficits, and self-awareness issues [20]. Aguilera *et al*. [21] found that individuals with the *Val66Met* polymorphism are more susceptible to depression, with *Met* allele carriers experiencing depressive episodes at an early age than those with the *Val/Val* genotype. Moreover, the *Met* allele has been associated with a higher risk of suicide in female patients, indicating its potential role as a predisposing factor for depression [22, 23]. The BDNF *Val66Met* polymorphism is linked to antidepressant efficacy [24, 25, 26]. Studies, including those by Alexopoulos *et al*. [24], reported that older patients with the *Met* allele showed improved therapeutic outcomes after 12 weeks of treatment with escitalopram at 10 mg/day compared to those with other genotypes. Another study found an 82% higher remission rate in *Met* allele carriers after 6 months of antidepressant treatment than in* Val/Val* carriers [25]. A meta-analysis of Asian populations similarly concluded that *Val/Met* carriers responded better to antidepressants than *Val/Val* carriers [26]. In contrast, a prior study within the Caucasian population did not identify a significant association between the BDNF *Val66Met* polymorphism and remission rates [27]. Thus, racial factors may need to be considered when addressing this issue.

People with major depressive disorder (MDD) have lower peripheral and central BDNF levels compared to non-depressed individuals [28]. Moreover, increased serum BDNF levels following antidepressant therapy correlate with symptom improvement [29]. Elevated BDNF levels are observed in responders and those who achieve remission, while levels remain stable in non-responders [30]. However, the relationship between serum BDNF levels and antidepressant efficacy in LLD remains contentious, potentially due to genetic polymorphisms across different populations [31]. The BDNF *Val66Met* polymorphism affects the presentation, progression, and prognosis of depression [32]. Serum BDNF levels were hypothesized to be associated with antidepressant efficacy in patients with LLD, and the BDNF *Val66Met* polymorphism influences this response. An open, prospective trial was conducted to verify this hypothesis by assessing the post-treatment changes in BDNF levels in patients with first-episode LLD. The association between the BDNF *Val66Met* polymorphism and the efficacy and cognitive impact of antidepressant treatment in patients with LLD after 8 weeks was aimed to be determined.

## 2. Materials and Methods

### 2.1 Participants

Patients were recruited from Beijing Anding Hospital, Capital Medical University, Beijing, China, between April 2020 and August 2022. The inclusion criteria were: first-episode patients diagnosed with depressive episodes, following the Diagnostic and Statistical Manual of Mental Disorders, 5th Edition (DSM-5); age of onset ≥60 years; at least 6 years of education (evidence indicates that educational attainment has positive effects on cognitive function) [33]; a score of ≥17 on the 17-item Hamilton Rating Scale for Depression Scale (HAMD-17); and a Mini-Mental State Examination (MMSE) score ≥24. Exclusion criteria included severe or unstable medical conditions affecting cardiovascular, urogenital, respiratory, endocrine, hematological, or nervous systems; depression secondary to other psychiatric disorders, physical illnesses, bipolar disorder, or rapid cycling; and alcohol or substance abuse or dependence. Furthermore, to ensure participant safety, we excluded individuals exhibiting severe suicidal tendencies, defined by HAMD-17 Item 3 [suicide] score ≥3, in accordance with recommendations from a previous study [34].

### 2.2 Treatment and Efficacy Assessment

All participants underwent clinical assessments and neurocognitive tests at baseline and after 8 weeks of treatment. As per the Chinese guidelines for the prevention and treatment of depressive disorders, treatment involved either escitalopram (5–15 mg/day, H. Lundbeck A/S, Copenhagen, Denmark) or sertraline (25–150 mg/day, Pfizer Inc., New York City, NY, USA), with dosages within the safe and effective range [35]. Patients experiencing severe sleep disturbances, anxiety, or agitation may receive short-term treatment with benzodiazepines. Throughout the 8-week treatment period, none of the patients underwent physical therapies such as electroconvulsive therapy or transcranial magnetic stimulation therapy.

Treatment efficacy is frequently assessed clinically by response (a ≥50% reduction from baseline in the total score) or remission (a total HAMD-17 score ≤7). This study focused on significant changes in HAMD-17 scores pre- and post-treatment. Consequently, treatment effectiveness was defined as a reduction of ≥75% in the HAMD-17 score, following recommendations from previous research [36]. The percentage (%) change in HAMD-17 scores is calculated as follows: 100% × (Pre-treatment HAMD scores – Post-treatment HAMD scores)/Pre-treatment HAMD scores. A negative percentage indicates worsening depressive symptoms.

### 2.3 Neurocognitive Assessment

Neurocognitive function was assessed using the Repeatable Battery for the Assessment of Neuropsychological Status (RBANS) [37], which includes 12 tasks covering five cognitive domains: (a) Immediate Memory, including verbal learning and story memory. (b) Visuospatial/Constructional, involving figure copy and line orientation tasks. (c) Language, with picture naming and semantic fluency tasks. (d) Attention, comprising digit span and coding tasks. (e) Delayed Memory, encompassing verbal (recall and recognition), story, and visual memory tasks. The MMSE was employed for inclusion and exclusion criteria. The RBANS total score, derived from summing the five index scores, typically ranges from 90 to 109, with lower scores indicating diminished cognitive function. A prior study indicated that except for the Delayed Memory Index, the proportion of variance accounted for by age is too small to merit clinical adjustment of index scores in a nondemented geriatric sample [38]. In addition, the RBANS is simple to administer and normally takes approximately 20 minutes. In short, the application of RBANS to assess the cognitive function of patients with LLD is feasible, and aligns with the findings of prior studies [35, 39].

### 2.4 Quality Control

Two professional psychiatrists assessed all scales. Before the start of the research, all participants underwent rigorous and comprehensive consistency training. The entire process adhered strictly to the experimental protocol. Researchers meticulously completed the case report forms, maintained accurate records, and refrained from making unauthorized changes. After each visit and evaluation, original data were promptly verified and reviewed to address any issues, ensuring the experimental data’s authenticity, accuracy, and completeness.

### 2.5 Measurement of Serum BDNF Concentration

Serum BDNF concentrations were measured before and after 8 weeks of treatment to evaluate BDNF levels in patients. Eligible participants who provided informed consent had 4 mL of venous blood collected from the forearm: 2 mL in ethylenediaminetetraacetic acid (EDTA) and 2 mL in heparin, and were immediately placed on ice. Blood for BDNF assays was collected in serum separator tubes, allowed to clot at room temperature for 1 h, and then subjected to platelet activation for 1 h at 4 °C. The blood was centrifuged at 2000 g for 20 min, and the serum was separated and stored at –80 °C until analysis. Serum BDNF levels were measured using an Enzyme-Linked Immunosorbent Assay (ELISA) with a BDNF sandwich ELISA kit (DuoSet; R&D Systems, Minneapolis, MN, USA). All the samples were tested in duplicate. The procedure involved diluting capture antibodies (provided by the manufacturer) in phosphate-buffered saline (PBS) (DuoSet; R&D Systems, Minneapolis, MN, USA), adding them to each well, and incubating overnight at room temperature. Plates were washed four times with PBS containing 0.05% Tween 20, blocked with 1% ovine Serum Albumin (BSA) (DuoSet; R&D Systems, Minneapolis, MN, USA) for 1 hour, and washed again four times with PBS and 0.05% Tween 20. Detection antibodies (provided by the manufacturer) were added and incubated for 2 hours. After washing, the plates were incubated with streptavidin-HRP (DuoSet) and developed with TMB chromogenic substrate (Kirkegaard & Perry Laboratories [KPL], Milford, MA, USA) for 15 minutes in the dark. The reaction was stopped with 1 M phosphoric acid, and absorbance was measured at 450 nm using a plate reader (Infinite M1000 PRO; Tecan Trading AG, Männedorf, Zurich, Switzerland).

### 2.6 DNA Extraction and Genetic Polymorphism Determination

Genomic DNA was extracted from peripheral whole blood samples using the G-DEX™ II Genomic DNA Extraction Kit (Intron Biotechnology, Seoul, South Korea), following the manufacturer’s protocol. Primer extension products generated from genomic DNA were assessed using matrix-assisted laser desorption/ionization time-of-flight (MALDI-TOF) mass spectrometry-based chip technology to analyze the BDNF (*Val66Met*) gene polymorphism. Data were processed with MassArray Typer 4.1 software (Agena Bioscience, San Diego, CA, USA), facilitating SNP typing by evaluating molecular size differences. The upstream primer for BDNF *Val66Met* polymorphism is 5^′^-ACTCTGGAGACGTGATGG-3^′^, and the downstream primer is 5^′^-ACTACTGAGCATCACCCTGGA-3^′^. Polymerase chain reaction (PCR) conditions included an initial denaturation at 95 °C for 5 minutes, followed by 40 cycles of denaturation at 95 °C for 30 seconds, annealing at 62 °C for 30 seconds, extension at 72 °C for 30 seconds, a final extension step at 72 °C for 5 minutes, and maintenance at 4 °C. PCR products were digested with the restriction enzyme Eco72I (New England Biolabs [NEB], Ipswich, MA, USA) at 37 °C, and gel electrophoresis was used to detect the 196G (*Val*, 99- and 72-bp fragments) and 196A (*Met*, 171-bp fragment) alleles. Based on the digestion pattern, genotypes were classified as *Val/Val*, *Val/Met*, or *Met/Met*.

### 2.7 Statistical Analysis

Statistical analyses were performed using Statistical Package for the Social Sciences version 22.0 (IBM SPSS Corp.; Armonk, NY, USA). The normality of data distribution was assessed via skewness and kurtosis. Continuous variables such as age and disease duration were presented as mean ± standard deviation, while categorical variables were reported as frequencies and percentages. Parametric data were analyzed using *t*-tests, Wilcoxon tests, or Kruskal–Wallis tests, whereas categorical data were evaluated with chi-square tests. Binary logistic regression explored relationships between BDNF concentration, its gene polymorphism, cognitive function, and therapeutic outcomes, adjusting for demographic factors. All tests were two-tailed, with significance set at *p* < 0.05.

## 3. Results

### 3.1 Demographic Data

**Fig. 1.**
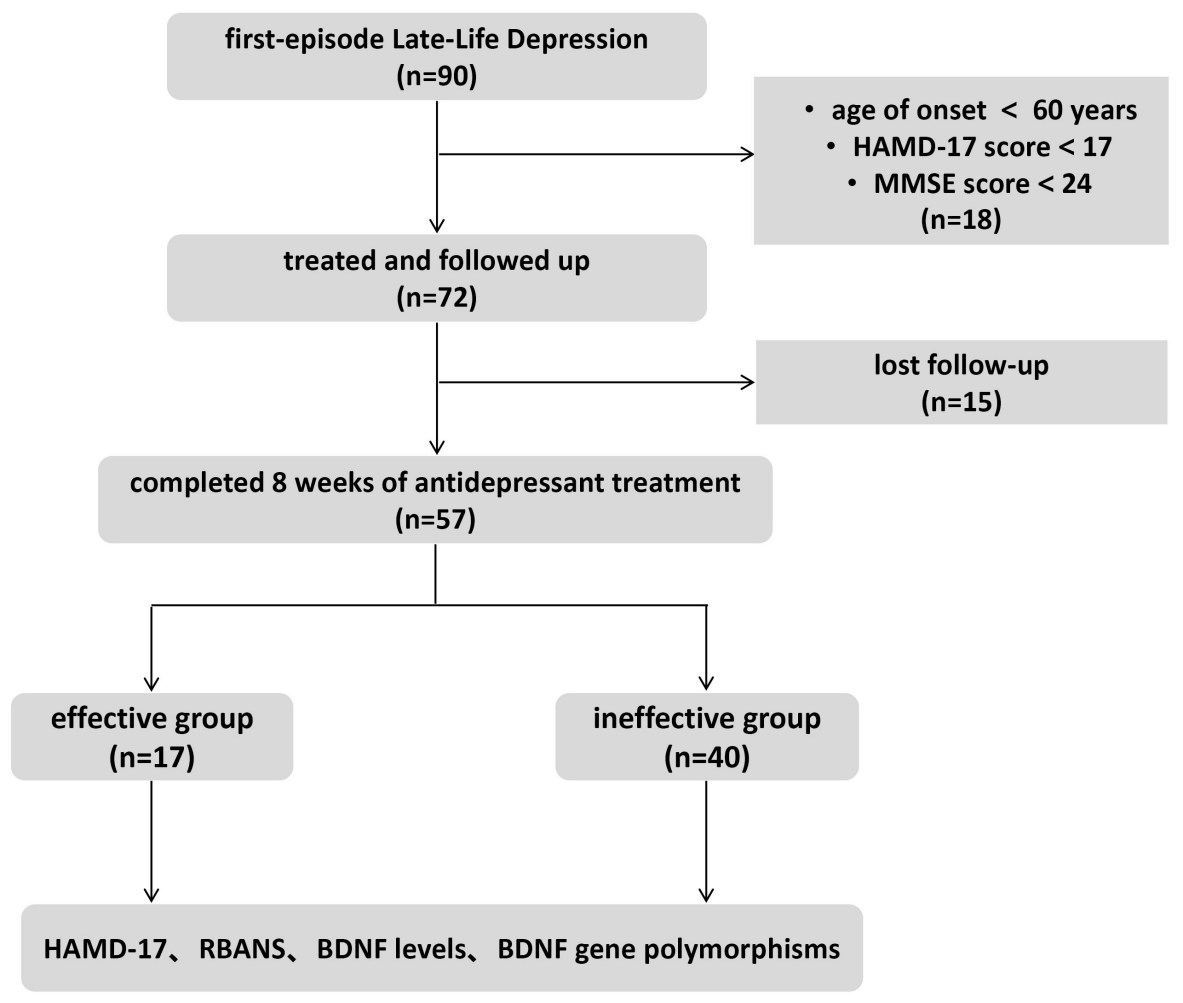
**Enrollment flow chart**. HAMD-17, 17-item Hamilton Depression Rating Scale; MMSE, Mini-Mental State Examination; RBANS, Repeatable Battery for the Assessment of Neuropsychological Status; BDNF, brain-derived neurotrophic factor.

Initially, 90 patients were screened for the study, of whom 72 met the inclusion criteria. However, 15 patients withdrew before completing the 8-week treatment and follow-up assessments, leaving 57 patients in the final cohort (Fig. 1). The average age of participants was 68.8 ± 4.7 years, with an average disease duration of 18.4 ± 25.5 months. The cohort included 19 male patients and 38 female patients. Among these, 15 patients (26.3%) carried the *Met/Met* genotype, 29 (50.9%) had the *Met/Val *genotype, and 13 (22.8%) had the *Val/Val* genotype. Significant differences were found between the effective and ineffective groups regarding sex (*p* = 0.041) and BDNF gene polymorphism (*p* = 0.013). However, no considerable differences were observed between the groups regarding age, marital status, education level, occupation, and disease duration (*p* > 0.05; Table 1).

**Table 1.** **Comparison of demographic characteristics between the effective and ineffective treatment groups (*n* = 57)**.

Variable		Mean ± SD or n (%)	Total patients (*n* = 57)		*T/χ^2^*	*p*
			Effective group (*n* = 17)	Ineffective group (*n* = 40)		
Age (years)		68.8 ± 4.7	70.4 ± 4.1	68.1 ± 4.8	1.710	0.093
Marital status, * n (%)*					0.525	1.290
	Married (Living with spouse)	23 (40.4)	6 (26.1)	17 (72.9)		
	Married (Living alone)	24 (42.1)	9 (52.9)	15 (37.5)		
	Widowed	10 (17.5)	2 (11.8)	8 (20.0)		
Sex, *n (%)*					4.191	0.041*
	Female	38 (66.7)	8 (21.1)	30 (78.9)		
	Male	19 (33.3)	9 (47.4)	10 (52.6)		
Education, *n (%)*					0.007	0.934
	Junior high school and below	34 (59.6)	10 (29.4)	24 (70.6)		
	High school and above	23 (40.4)	7 (30.4)	16 (69.6)		
Occupation, *n (%)*					0.068	0.794
	Informal	35 (61.4)	10 (28.6)	25 (71.4)		
	Formal	22 (38.6)	7 (31.8)	15 (68.2)		
Disease duration (months)		18.4 ± 25.5			1.417	0.234
	<10	27 (47.4)	6 (22.2)	21 (77.8)		
	≥10	30 (52.6)	11 (36.7)	19 (63.3)		
BDNF gene polymorphism					8.644	0.013*
	*Met/Met*	15 (26.3)	2 (13.3)	13 (86.7)		
	*Met/Val*	29 (50.9)	7 (24.1)	22 (75.9)		
	*Val/Val*	13 (22.8)	8 (61.5)	5 (38.5)		

*p < 0.05. BDNF, brain-derived neurotrophic factor; SD, standard deviation.

### 3.2 Changes in HAMD-17 Scores, RBANS Scores, and Serum BDNF Levels before and after Treatment

Before treatment, the HAMD-17 score averaged 21.8 ± 3.7, which decreased to 8.6 ± 5.7 after 8 weeks. This difference was significant (*p* = 0.001). The RBANS score at baseline was 146.7 ± 29.0, rising to 155.4 ± 29.0 after 8 weeks, with a substantial difference (*p* < 0.001). Serum BDNF levels increased from 31.50 ± 15.20 ng/mL before treatment to 38.46 ± 18.46 ng/mL after 8 weeks, showing a significant change (*p* = 0.026; Table 2).

**Table 2.** **Changes in HAMD-17 scores, RBANS scores, and serum BDNF levels before and after treatment**.

Project	Before treatment	After treatment	*t*	*p*
HAMD-17	21.80 ± 3.70	8.60 ± 5.70	15.522	0.001*
RBANS	146.70 ± 29.00	155.4 ± 29.0	–2.287	<0.001*
Serum BDNF levels (ng/mL)	31.50 ± 15.20	38.46 ± 18.46	–4.461	0.026*

*p < 0.05. HAMD-17, 17-item Hamilton Depression Rating Scale; RBANS, Repeatable Battery for the Assessment of Neuropsychological Status.

### 3.3 Comparison of Cognitive Function Changes between the Effective and Ineffective Groups

At baseline, the total RBANS score for the effective group was 148.20 ± 27.46, compared to 143.12 ± 32.94 for the ineffective group. Significant differences in RBANS scores were observed before and after treatment in both groups (*p* < 0.05). Post-treatment, the effective group showed a change in RBANS scores of 9.76 ± 11.55, whereas the ineffective group exhibited a change of 8.28 ± 16.04. No significant difference in RBANS score changes from baseline was found between the two groups (*p* = 0.419). Furthermore, no significant differences were observed regarding changes in immediate memory, delayed memory, spatial construction, language function, or attention between the two groups (*p* > 0.05; Table 3).

**Table 3.** **Comparison of cognitive function changes between the effective and ineffective groups**.

Changes of RBANS	Effective group	Ineffective group	*t*	*p*
Total score	9.76 ± 11.55	8.28 ± 16.04	0.346	0.419
Immediate memory	3.00 ± 5.44	1.65 ± 6.40	0.760	0.450
Delayed memory	4.59 ± 6.19	2.88 ± 5.28	1.065	0.292
Spatial construction	0.12 ± 4.00	0.78 ± 7.32	0.348	0.729
Language function	1.71 ± 5.51	1.93 ± 4.45	0.158	0.875
Attention	2.29 ± 6.23	2.00 ± 9.94	–0.113	0.911

### 3.4 Changes in BDNF Levels before and after Treatment

At the end of the eighth week of treatment, BDNF levels were evaluated in both groups. In the effective group, the change in BDNF levels from baseline was 9626.39 ± 17,317.89 pg/mL, while in the ineffective group, it was 5833.13 ± 25,131.70 pg/mL. No significant difference in BDNF level changes from baseline was noted between the effective and ineffective groups (*p* = 0.573). However, within the effective group, a significant difference was observed in BDNF levels before and after treatment (*p* = 0.036); Tables 4,5.

**Table 4.** **Comparison of changes in BDNF levels before and after treatment between the effective and ineffective groups**.

Group	Difference	Difference 95% CI	*t*	*p*
Ineffective group	5833.13 ± 25,131.70	–3793.26 (–3805.75~–3780.78)	–0.57	0.573
Effective group	9626.39 ± 17,317.89			

**Table 5.** **Comparison of BDNF levels before and after treatment between the effective and ineffective groups**.

Group	Before treatment	After treatment	*t*	*p*
Ineffective group	32,907.85 ± 16,469.25	38,740.98 ± 20,205.36	–1.47	0.150
Effective group	28,176.70 ± 11,432.59	37,803.09 ± 14,019.18	–2.29	0.036*
*t*	1.08	0.17	-	-
*p*	0.286	0.863	-	-

*p < 0.05.

### 3.5 Logistic Regression Analysis of Treatment Efficacy

Treatment efficacy was analyzed as the dependent variable in a logistic regression model, incorporating age, sex, BDNF gene polymorphism, and changes in RBANS scores and BDNF levels. The analysis revealed that treatment efficacy was significantly associated with sex and the BDNF gene polymorphism *Val/Val* (Table 6).

**Table 6.** **Binary logistic regression analysis of treatment efficacy with sex and BDNF gene polymorphisms**.

Project	Wald χ^2^	OR	95% CI	*p*
Age	1.603	1.115	0.942–1.319	0.205
Sex	7.171	10.094	1.859–54.820	0.007*
Changes of RBANS	0.121	0.991	0.943–1.042	0.728
Changes of BDNF levels	0.258	1.000	1.000–1.000	0.611
BDNF gene polymorphism	9.002	6.559	1.920–22.408	0.003*

*p < 0.05.

## 4. Discussion

This study explored the impact of an 8-week antidepressant treatment on serum BDNF levels in older patients experiencing first-episode major depression. It also examined the association between BDNF* Val66Met* gene polymorphism, treatment efficacy, and cognitive function. The results revealed that after 8 weeks of antidepressant treatment, Han Chinese patients with first-episode LLD exhibited significant improvements in depressive symptoms, increased serum BDNF levels, and enhanced cognitive function. The effective and ineffective groups found no significant differences in RBANS scores or serum BDNF levels. Binary logistic regression identified male sex and BDNF gene polymorphism as predictors of treatment efficacy.

BDNF, a protein distributed throughout the central nervous system, plays a vital role by interacting with the tyrosine kinase receptor B (TrkB), which supports neurogenesis, neuronal survival, differentiation, and plasticity [40]. Decreased serum BDNF levels are associated with a higher risk of depression, and antidepressant treatment can modulate these levels, thereby exerting antidepressant effects [41, 42, 43]. Our study found lower serum BDNF concentrations in patients with LLD, which increased considerably after 8 weeks of treatment. Similarly, Wolkowitz *et al*. [44] found lower baseline BDNF levels in depressed participants, which significantly increased after 8 weeks of treatment with escitalopram or sertraline, regardless of depression severity. A study involving 40 patients with LLD treated with paroxetine revealed lower baseline BDNF levels compared to the general population, normalizing after effective treatment, with minimal changes in the ineffective treatment group [45]. This observation suggests a potential link between increased serum BDNF levels and improved depressive symptoms. A meta-analysis indicated that various antidepressants have differing effects on BDNF levels [46], with sertraline showing a superior early increase in BDNF concentrations compared to other drugs (such as venlafaxine, paroxetine, or escitalopram) [46], This result underscores the value of further examining the relationship between BDNF and antidepressant pharmacology in peripheral blood. Despite the significant increase in BDNF levels and reduction in HAMD-17 scores observed in our study, no direct correlation was identified between BDNF level changes and treatment efficacy. Previous studies have reported increased BDNF levels in treatment responders or remitters but not in non-responders [30]. However, in this study, no significant differences in BDNF level changes were observed between the effective and ineffective treatment groups, which may be attributed to the small sample size.

Research on BDNF genetic polymorphisms, particularly *Val66Met*, often yields conflicting results. Aldoghachi *et al*. [47] examined three BDNF gene variants (*rs6265*, *rs1048218*, *rs1048220*) in 300 Malaysian participants with depression. The homozygous *Met/Met* genotype of rs6265 increased depression risk by 1.75 times compared to the *Val/Val* genotype. Similar findings were reported by Ribeiro *et al*. [48] in Caucasians and a study in Taiwan [49], both indicating an increased depression risk associated with the *Met/Met *genotype. However, Terracciano *et al*. [50] reported lower serum BDNF levels in depressed patients compared to non-depressed controls, with no significant association between BDNF *Val66Met* genotype and depression risk or serum BDNF levels. These results suggest that lower serum BDNF levels correlate with depression; the BDNF* Val66Met* genotype may not significantly influence this relationship.

Depression is associated with structural brain abnormalities in regions such as the prefrontal cortex, cingulate cortex, hippocampus, and amygdala [51]. Fractional anisotropy (FA), a measure of neuronal fiber integrity, reflects these structural changes [52]. Studies have explored genetic and environmental factors influencing FA and depression. For instance, BDNF *Val66Met* gene variations affect the integrity of the uncinate fasciculus (UF), with depressed individuals carrying the Met allele showing lower FA in the UF [20, 53]. Furthermore, the BDNF *Val66Met* polymorphism moderates the correlation between FA in the UF and depression severity [54]. Tatham *et al*. [55] demonstrated that antidepressant effects on the left UF related to BDNF genotype, with *Val/Val* carriers frequently exhibiting better FA and treatment response. These findings suggest that genetic influences on brain connectivity may influence antidepressant outcomes. Moreover, psychosocial factors, such as childhood adversity, significantly impact neural structure and interact with the BDNF *Val66Met* polymorphism [56, 57]. Jaworska *et al*. [58] reported no effect of the BDNF *Val66Met* variant on cortical thickness or hippocampal volume, aligning with other studies. However, Cao *et al*. [59] reported reduced hippocampal volume in Met allele carriers, consistent with previous research. Despite ongoing debate, it is clear that BDNF influences the structure of various brain regions, underscoring the need for further investigation into its role in the central nervous system.

The relationship between BDNF and antidepressant response is complex. Depression involves multiple neurobiological pathways, including the dopaminergic, noradrenergic, glutamatergic, and serotonergic systems, as well as inflammatory markers [60]. BDNF is crucial in mediating neuronal changes that contribute to symptom improvement during antidepressant treatment. Increased serum BDNF levels are frequently observed in patients undergoing treatment, suggesting that BDNF may serve as a potential biomarker for Selective Serotonin Reuptake Inhibitor (SSRI) response, given its involvement in the serotonin system [61]. However, there are conflicting reports on the long-term impact of antidepressant use on serum BDNF levels. Branchi [62] proposes that BDNF may only be one component of the broader, multifaceted effects of antidepressants, which may enhance brain plasticity rather than directly improve mood.

Depression is known to be nearly twice as prevalent in women as in men [63], and symptomatic profiles differ significantly between the sexes [64]. However, findings on sex differences in treatment response are less consistent, with many studies reporting no significant variation between men and women. Wilson *et al*. [65] assessed the role of sex in the relationship between acute functional connectivity changes (measured by functional Magnetic Resonance Imaging [fMRI]) and treatment response in LLD. Their findings indicated differences in one-day connectivity changes between remitters and non-remitters in men but not women. Our study suggests that male patients with LLD may benefit more from an 8-week antidepressant treatment. Further research should focus on segmenting male patients to enhance personalized and precise treatment strategies.

This study has several limitations. First, the absence of a healthy control group limits the ability to fully understand variations in BDNF levels among older patients with depression. Specifically, whether post-treatment BDNF levels differ from those of healthy controls remains unexplored. Second, the absence of a single medication and the small sample size may affect the interpretation of clinical significance. Third, the short observational period of only 8 weeks restricted data collection on long-term cognitive function and BDNF level changes, thereby hindering the observation of short- and long-term treatment effects. Lastly, our findings are based on strong antidepressant responses, defining treatment effectiveness as a 75% reduction in the HAMD-17 score is unusual and may lack clinical clarity, affecting comparability and generalizability of the findings.

## 5. Conclusion

In conclusion, antidepressant treatment for 8 weeks altered serum BDNF levels in patients with LLD, with male patients carrying the *Val/Val* genotype potentially responding better to conventional antidepressants.

## Availability of Data and Materials

The data that support the findings of this study are available from the corresponding author upon reasonable request.

## References

[1] Alexopoulos GS (2005). Depression in the elderly. *Lancet (London, England)*.

[2] Gambaro E, Gramaglia C, Azzolina D, Campani D, Molin AD, Zeppegno P (2022). The complex associations between late life depression, fear of falling and risk of falls. A systematic review and meta-analysis. *Ageing Research Reviews*.

[3] Piel C, Quante A (2023). Therapy Strategies for Late-life Depression: A Review. *Journal of Psychiatric Practice*.

[4] Cai W, Ma W, Mueller C, Stewart R, Ji J, Shen WD (2023). Association between late-life depression or depressive symptoms and stroke morbidity in elders: A systematic review and meta-analysis of cohort studies. *Acta Psychiatrica Scandinavica*.

[5] Linnemann C, Lang UE (2020). Pathways Connecting Late-Life Depression and Dementia. *Frontiers in Pharmacology*.

[6] Leyhe T, Reynolds CF, Melcher T, Linnemann C, Klöppel S, Blennow K (2017). A common challenge in older adults: Classification, overlap, and therapy of depression and dementia. Alzheimer’s & Dementia: the Journal of the Alzheimer’s Association. *Alzheimer’s & Dementia: the Journal of the Alzheimer’s Association*.

[7] Beekman AT, Deeg DJ, Geerlings SW, Schoevers RA, Smit JH, van Tilburg W (2001). Emergence and persistence of late life depression: a 3-year follow-up of the Longitudinal Aging Study Amsterdam. *Journal of Affective Disorders*.

[8] Borges S, Chen YF, Laughren TP, Temple R, Patel HD, David PA (2014). Review of maintenance trials for major depressive disorder: a 25-year perspective from the US Food and Drug Administration. *The Journal of Clinical Psychiatry*.

[9] Deng Y, McQuoid DR, Potter GG, Steffens DC, Albert K, Riddle M (2018). Predictors of recurrence in remitted late-life depression. *Depression and Anxiety*.

[10] Huang EJ, Reichardt LF (2001). Neurotrophins: roles in neuronal development and function. *Annual Review of Neuroscience*.

[11] Martinowich K, Manji H, Lu B (2007). New insights into BDNF function in depression and anxiety. *Nature Neuroscience*.

[12] Correia AS, Cardoso A, Vale N (2023). BDNF Unveiled: Exploring Its Role in Major Depression Disorder Serotonergic Imbalance and Associated Stress Conditions. *Pharmaceutics*.

[13] Yang T, Nie Z, Shu H, Kuang Y, Chen X, Cheng J (2020). The Role of BDNF on Neural Plasticity in Depression. *Frontiers in Cellular Neuroscience*.

[14] Chao MV (2003). Neurotrophins and their receptors: a convergence point for many signalling pathways. *Nature Reviews. Neuroscience*.

[15] Duman RS, Deyama S, Fogaça MV (2021). Role of BDNF in the pathophysiology and treatment of depression: Activity-dependent effects distinguish rapid-acting antidepressants. *The European Journal of Neuroscience*.

[16] Berton O, McClung CA, Dileone RJ, Krishnan V, Renthal W, Russo SJ (2006). Essential role of BDNF in the mesolimbic dopamine pathway in social defeat stress. *Science (New York, N.Y.)*.

[17] Vega JA, García-Suárez O, Hannestad J, Pérez-Pérez M, Germanà A (2003). Neurotrophins and the immune system. *Journal of Anatomy*.

[18] Januar V, Ancelin ML, Ritchie K, Saffery R, Ryan J (2015). BDNF promoter methylation and genetic variation in late-life depression. *Translational Psychiatry*.

[19] Yin Y, Hou Z, Wang X, Sui Y, Yuan Y (2015). The BDNF Val66Met polymorphism, resting-state hippocampal functional connectivity and cognitive deficits in acute late-onset depression. *Journal of Affective Disorders*.

[20] Carballedo A, Amico F, Ugwu I, Fagan AJ, Fahey C, Morris D (2012). Reduced fractional anisotropy in the uncinate fasciculus in patients with major depression carrying the met-allele of the Val66Met brain-derived neurotrophic factor genotype. *American Journal of Medical Genetics. Part B, Neuropsychiatric Genetics: the Official Publication of the International Society of Psychiatric Genetics*.

[21] Aguilera M, Arias B, Wichers M, Barrantes-Vidal N, Moya J, Villa H (2009). Early adversity and 5-HTT/BDNF genes: new evidence of gene-environment interactions on depressive symptoms in a general population. *Psychological Medicine*.

[22] Kanellopoulos D, Gunning FM, Morimoto SS, Hoptman MJ, Murphy CF, Kelly RE (2011). Hippocampal volumes and the brain-derived neurotrophic factor val66met polymorphism in geriatric major depression. *The American Journal of Geriatric Psychiatry: Official Journal of the American Association for Geriatric Psychiatry*.

[23] Liu RJ, Lee FS, Li XY, Bambico F, Duman RS, Aghajanian GK (2012). Brain-derived neurotrophic factor Val66Met allele impairs basal and ketamine-stimulated synaptogenesis in prefrontal cortex. *Biological Psychiatry*.

[24] Alexopoulos GS, Glatt CE, Hoptman MJ, Kanellopoulos D, Murphy CF, Kelly RE (2010). BDNF val66met polymorphism, white matter abnormalities and remission of geriatric depression. *Journal of Affective Disorders*.

[25] Taylor WD, McQuoid DR, Ashley-Koch A, MacFall JR, Bridgers J, Krishnan RR (2011). BDNF Val66Met genotype and 6-month remission rates in late-life depression. *The Pharmacogenomics Journal*.

[26] Zou YF, Ye DQ, Feng XL, Su H, Pan FM, Liao FF (2010). Meta-analysis of BDNF Val66Met polymorphism association with treatment response in patients with major depressive disorder. *European Neuropsychopharmacology: the Journal of the European College of Neuropsychopharmacology*.

[27] Wilkie MJV, Smith D, Reid IC, Day RK, Matthews K, Wolf CR (2007). A splice site polymorphism in the G-protein beta subunit influences antidepressant efficacy in depression. *Pharmacogenetics and Genomics*.

[28] Cavaleri D, Moretti F, Bartoccetti A, Mauro S, Crocamo C, Carrà G (2023). The role of BDNF in major depressive disorder, related clinical features, and antidepressant treatment: Insight from meta-analyses. *Neuroscience and Biobehavioral Reviews*.

[29] Kishi T, Yoshimura R, Ikuta T, Iwata N (2018). Brain-Derived Neurotrophic Factor and Major Depressive Disorder: Evidence from Meta-Analyses. *Frontiers in Psychiatry*.

[30] Polyakova M, Stuke K, Schuemberg K, Mueller K, Schoenknecht P, Schroeter ML (2015). BDNF as a biomarker for successful treatment of mood disorders: a systematic & quantitative meta-analysis. *Journal of Affective Disorders*.

[31] Zhao M, Chen L, Yang J, Han D, Fang D, Qiu X (2018). BDNF Val66Met polymorphism, life stress and depression: A meta-analysis of gene-environment interaction. *Journal of Affective Disorders*.

[32] Björkholm C, Monteggia LM (2016). BDNF - a key transducer of antidepressant effects. *Neuropharmacology*.

[33] Lövdén M, Fratiglioni L, Glymour MM, Lindenberger U, Tucker-Drob EM (2020). Education and Cognitive Functioning Across the Life Span. *Psychological Science in the Public Interest: a Journal of the American Psychological Society*.

[34] Cui J, Wang Y, Liu R, Chen X, Zhang Z, Feng Y (2021). Effects of escitalopram therapy on resting-state functional connectivity of subsystems of the default mode network in unmedicated patients with major depressive disorder. *Translational Psychiatry*.

[35] Liu C, Li L, Pan W, Zhu D, Lian S, Liu Y (2023). Altered topological properties of functional brain networks in patients with first episode, late-life depression before and after antidepressant treatment. *Frontiers in Aging Neuroscience*.

[36] Leucht S, Fennema H, Engel R, Kaspers-Janssen M, Lepping P, Szegedi A (2013). What does the HAMD mean?. *Journal of Affective Disorders*.

[37] Goette WF, Goette HE (2019). A meta-analysis of the accuracy of embedded performance validity indicators from the repeatable battery for the assessment of neuropsychological status. *The Clinical Neuropsychologist*.

[38] Gontkovsky ST, Mold JW, Beatty WW (2002). Age and educational influences on RBANS index scores in a nondemented geriatric sample. *The Clinical Neuropsychologist*.

[39] Pan W, Liu C, Zhu D, Liu Y, Mao P, Ren Y (2022). Prediction of Antidepressant Efficacy by Cognitive Function in First-Episode Late-Life Depression: A Pilot Study. *Frontiers in Psychiatry*.

[40] Rantamäki T, Castrén E (2008). Targeting TrkB neurotrophin receptor to treat depression. *Expert Opinion on Therapeutic Targets*.

[41] Knöchel C, Alves G, Friedrichs B, Schneider B, Schmidt-Rechau A, Wenzler S (2015). Treatment-resistant Late-life Depression: Challenges and Perspectives. *Current Neuropharmacology*.

[42] Shirayama Y, Chen ACH, Nakagawa S, Russell DS, Duman RS (2002). Brain-derived neurotrophic factor produces antidepressant effects in behavioral models of depression. *The Journal of Neuroscience: the Official Journal of the Society for Neuroscience*.

[43] Adachi M, Barrot M, Autry AE, Theobald D, Monteggia LM (2008). Selective loss of brain-derived neurotrophic factor in the dentate gyrus attenuates antidepressant efficacy. *Biological Psychiatry*.

[44] Wolkowitz OM, Wolf J, Shelly W, Rosser R, Burke HM, Lerner GK (2011). Serum BDNF levels before treatment predict SSRI response in depression. *Progress in Neuro-psychopharmacology & Biological Psychiatry*.

[45] Li JM, Yan JD, Deng YQ (2012). Changes and clinical correlation of serum BDNF levels in elderly patients with depression before and after treatment with paroxetine. *Guide of China Medicine*.

[46] Zhou C, Zhong J, Zou B, Fang L, Chen J, Deng X (2017). Meta-analyses of comparative efficacy of antidepressant medications on peripheral BDNF concentration in patients with depression. *PloS One*.

[47] Aldoghachi AF, Tor YS, Redzun SZ, Lokman KAB, Razaq NAA, Shahbudin AF (2019). Screening of brain-derived neurotrophic factor (BDNF) single nucleotide polymorphisms and plasma BDNF levels among Malaysian major depressive disorder patients. *PloS One*.

[48] Ribeiro L, Busnello JV, Cantor RM, Whelan F, Whittaker P, Deloukas P (2007). The brain-derived neurotrophic factor rs6265 (Val66Met) polymorphism and depression in Mexican-Americans. *Neuroreport*.

[49] Hwang JP, Tsai SJ, Hong CJ, Yang CH, Lirng JF, Yang YM (2006). The Val66Met polymorphism of the brain-derived neurotrophic-factor gene is associated with geriatric depression. *Neurobiology of Aging*.

[50] Terracciano A, Piras MG, Lobina M, Mulas A, Meirelles O, Sutin AR (2013). Genetics of serum BDNF: meta-analysis of the Val66Met and genome-wide association study. *The World Journal of Biological Psychiatry: the Official Journal of the World Federation of Societies of Biological Psychiatry*.

[51] Stahl SM (2013). The last Diagnostic and Statistical Manual (DSM): replacing our symptom-based diagnoses with a brain circuit-based classification of mental illnesses. *CNS Spectrums*.

[52] Lee J, Ju G, Park H, Chung S, Son JW, Shin CJ (2022). Hippocampal Subfields and White Matter Connectivity in Patients with Subclinical Geriatric Depression. *Brain Sciences*.

[53] Han KM, Choi S, Kim A, Kang J, Won E, Tae WS (2018). The effects of 5-HTTLPR and BDNF Val66Met polymorphisms on neurostructural changes in major depressive disorder. *Psychiatry Research. Neuroimaging*.

[54] Tatham EL, Ramasubbu R, Gaxiola-Valdez I, Cortese F, Clark D, Goodyear B (2016). White matter integrity in major depressive disorder: Implications of childhood trauma, 5-HTTLPR and BDNF polymorphisms. *Psychiatry Research. Neuroimaging*.

[55] Tatham EL, Hall GBC, Clark D, Foster J, Ramasubbu R (2017). The 5-HTTLPR and BDNF polymorphisms moderate the association between uncinate fasciculus connectivity and antidepressants treatment response in major depression. *European Archives of Psychiatry and Clinical Neuroscience*.

[56] Meinert S, Repple J, Nenadic I, Krug A, Jansen A, Grotegerd D (2019). Reduced fractional anisotropy in depressed patients due to childhood maltreatment rather than diagnosis. *Neuropsychopharmacology: Official Publication of the American College of Neuropsychopharmacology*.

[57] Tendolkar I, Mårtensson J, Kühn S, Klumpers F, Fernández G (2018). Physical neglect during childhood alters white matter connectivity in healthy young males. *Human Brain Mapping*.

[58] Jaworska N, MacMaster FP, Foster J, Ramasubbu R (2016). The influence of 5-HTTLPR and Val66Met polymorphisms on cortical thickness and volume in limbic and paralimbic regions in depression: a preliminary study. *BMC Psychiatry*.

[59] Cao B, Bauer IE, Sharma AN, Mwangi B, Frazier T, Lavagnino L (2016). Reduced hippocampus volume and memory performance in bipolar disorder patients carrying the BDNF val66met met allele. *Journal of Affective Disorders*.

[60] Diniz BS, Mulsant BH, Reynolds CF, Blumberger DM, Karp JF, Butters MA (2022). Association of Molecular Senescence Markers in Late-Life Depression With Clinical Characteristics and Treatment Outcome. *JAMA Network Open*.

[61] Flores-Ramos M, Vega-Rosas A, Palomera-Garfias N, Saracco-Alvarez R (2024). Are BDNF and Stress Levels Related to Antidepressant Response?. *Int J Mol Sci*.

[62] Branchi I (2009). The mouse communal nest: investigating the epigenetic influences of the early social environment on brain and behavior development. *Neuroscience and Biobehavioral Reviews*.

[63] Salk RH, Hyde JS, Abramson LY (2017). Gender differences in depression in representative national samples: Meta-analyses of diagnoses and symptoms. *Psychological Bulletin*.

[64] Eid RS, Gobinath AR, Galea LAM (2019). Sex differences in depression: Insights from clinical and preclinical studies. *Progress in Neurobiology*.

[65] Wilson JD, Gerlach AR, Karim HT, Aizenstein HJ, Andreescu C (2023). Sex matters: acute functional connectivity changes as markers of remission in late-life depression differ by sex. *Molecular Psychiatry*.

